# Effect of a Proton Pump Inhibitor on the Duodenum Microbiome of Gastric Ulcer Patients

**DOI:** 10.3390/life12101505

**Published:** 2022-09-27

**Authors:** Jung-Hyun Lim, Jongbeom Shin, Jin-Seok Park

**Affiliations:** Digestive Disease Center, Department of Internal Medicine, Inha University School of Medicine, Incheon 22332, Korea

**Keywords:** microbiota, duodenum, biopsy, PPI

## Abstract

The gut microbiota are regarded as a functional organ that plays a substantial role in human health and disease. Proton pump inhibitors (PPIs) are widely used in medicine but can induce changes in the overall gut microbiome and cause disease-associated dysbiosis. The microbiome of the duodenum has not been sufficiently studied, and the effects of PPIs on the duodenal microbiome are poorly understood. In this study, we investigated the effect of PPI administration on duodenum microbiota in patients with a gastric ulcer. A total of 12 gastric ulcer patients were included, and PPI (Ilaprazole, Noltec^®^, 10 mg) was prescribed in all patients for 4 weeks. A total of 17 samples from the second portion of the duodenum were analyzed. Microbiome compositions were assessed by sequencing the V3–V4 region of the 16s rRNA gene (Miseq). Changes in microbiota compositions after 4 weeks of PPI treatment were analyzed. a-Diversity was higher after PPI treatment (*p* = 0.02, at Chao1 index), and β-diversity was significantly different after treatment (*p* = 0.007). Welch’s *t*-test was used to investigate changes in phyla, genus, and species level, and the abundance of *Akkermansia muciniphila*, belonging to the phylum *Verrucomicrobia*, and *Porphyromonas endodontalis*, belonging to the phylum *Bacteroidetes*, was significantly increased after treatment (*p* = 0.044 and 0.05). PPI administration appears to induce duodenal microbiome dysbiosis while healing gastric ulcers. Further large-scale studies on the effects of PPIs on the duodenal microbiome are required.

## 1. Introduction

PPIs are prescribed to treat several gastric acid-related diseases such as upper gastrointestinal bleeding, peptic ulcers, erosive esophagitis, gastroesophageal reflux, and certain functional dyspepsia subtypes [1,2,3,4]. A PPI is a drug that inhibits gastric acid secretion by covalently binding with activating H+K+-ATPase on the surface of parietal cells in the mucous membrane of the stomach [5,6]. When a PPI is administered, stomach pH rises rapidly to achieve the therapeutic effect. PPIs are recommended as a treatment of choice for peptic ulcers and have a strong therapeutic effect by suppressing gastric acid secretion and preventing gastrointestinal bleeding and perforation [7,8,9,10].

Gastric acid works to kill potential pathogens that might otherwise enter the digestive system from the oral cavity [11]. Nevertheless, the stomach is colonized by bacteria such as *Actinobacteria*, *Bacteroidetes*, *Firmicutes*, *Proteobacteria* (includes *H. pylori*), and *Fusobacteria* [12]. Recent studies on the gastric microbiome have shown that the diversity of these microbiomes is correlated with gastric cancer, peptic ulcer, and gastric inflammation [13,14,15,16]. PPIs can affect gastric microbiome diversity by directly targeting bacterial and fungal proton pumps or disrupting the normal gastric microenvironment by increasing gastric pH [17]. Bacteria living in the oropharynx and feces are more abundant in the gastric microbiome after PPI use [18]. Furthermore, recent studies have shown that PPIs can induce changes in the overall gut microbiome and cause dysbiosis, which is associated with disease states, such as hepatic encephalopathy or spontaneous bacterial peritonitis in cirrhotic patients, and small intestinal bacterial overgrowth [19,20,21,22,23]. According to these studies, PPI-induced bacterial overgrowth occurs due to reduced gastric acid exposure in the intestine and can increase mortality in cirrhotic patients [19,20,21,22,23].

The duodenum is the first part of the small intestine, where chyme from the stomach, bile acid, and pancreatic enzymes are mixed and processed to improve absorption in the jejunum, and it also secretes various neurotransmitters and hormones through interaction with the duodenal microbiome [24]. The gut microbiome plays an important role in maintaining physiologic homeostasis and is also considered to be an independent factor that can cause diseases [25,26]. According to a recent study, the anti-inflammatory effects of PPIs in the duodenum are due to reduced acid exposure and eosinophilia [27]. However, few studies have investigated the association between PPI use and changes in the duodenal microbiome.

Therefore, we performed next-generation sequencing (NGS)-based analysis by 16S sequencing on mucosal samples of the second portion of the duodenum to identify changes in the duodenal microbiome induced by 4 weeks of PPI treatment in patients with a gastric ulcer.

## 2. Materials and Methods

### 2.1. Study Subjects

**Figure 1:**
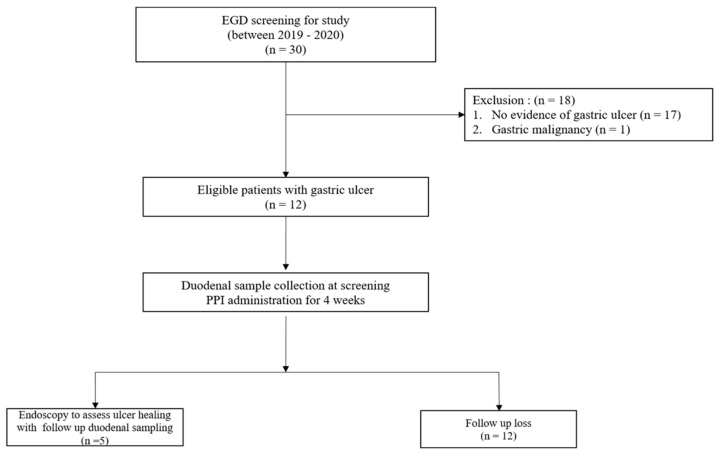
Flow diagram of the study.

Patients scheduled to undergo screening gastroscopy for the evaluation of epigastric pain at Inha University Hospital from March 2019 to February 2020 were recruited. Informed consent was obtained when a patient met the inclusion criteria. The inclusion criteria were as follows: (1) age > 18 years at the time of treatment, (2) gastric ulcer confirmed by endoscopy, (3) those who can take PPI treatment for more than 4 weeks, and (4) no evidence of dementia or cognitive impairment. The exclusion criteria were as follows: (1) allergic symptoms or hypersensitivity to PPIs such as ilaprazole, (2) those who have undergone treatment or surgery that may affect gastric acid secretion within 3 months of registration, (3) those who have taken antibiotics within 3 months of registration, (4) those who have taken probiotics within 1 month of registration, and (5) a pregnant status or breastfeeding. Thirty patients were screened, and of these, we excluded seventeen without evidence of gastric ulcer and one patient diagnosed with malignant gastric ulcer. Finally, 12 patients were enrolled (Figure 1). However, 7 of the 12 were lost to follow-up, and only 5 underwent a follow-up biopsy. The study was approved by the Institutional Review Board of Inha University Hospital, Incheon, South Korea (Approval number: 2020-01-029-009).

### 2.2. Study Procedure and Collection of Duodenal Samples

Duodenoscopy (GIF-HQ290, Olympus, Tokyo, Japan) was performed using scrupulously cleaned equipment according to standard endoscopic cleaning guidelines [28]. Sterile working channel plugs were used in all cases. Mucosal biopsies were all performed on the second portion of the duodenum.

Biopsies were performed using a standard endoscopic forceps. Each specimen was placed in a sterile tube and stored at −80 °C until required for analysis. After duodenoscopy the patients took ilaprazole (Noltec^®^, 10 mg) once daily for 4 weeks. After that, five patients underwent a follow-up biopsy on the second duodenal portion during endoscopy to confirm the healing of their gastric ulcer.

### 2.3. DNA Extraction from Duodenal Biopsy Samples

Whole bacterial genomic DNA extraction from biopsy tissue was performed using a Maxwell RSC PureFood GMO and Authentication Kit (Promega, Madison, WI, USA) following the manufacturer’s instruction. DNA concentrations were calculated using a UV-vis spectrophotometer (NanoDrop 2000c; Thermo Fisher Scientific, Waltham, MA, USA) and quantified using a QuantiFluor ONE dsDNA System (Promega). DNA samples were stored at −20 °C until required for experiments.

### 2.4. PCR Amplification of the V3–V4 Region of the Bacterial 16S Ribosomal RNA (rRNA) Gene

The V3–V4 region of the bacterial 16S rRNA gene was amplified from extracted DNA using the F319 (5′-TCGTCGGCAGCGTCAGATGTGTATAAGAGACAGCCTACG-GGNGGCWGCAG) and R806 (5′-GTCTCGTGGGCTCGGAGATGTGTATAAGAGACA-GGACTACHVGGGTATC-TAATCC-3′) primer sets. DNA templates (12.5 ng/uL) were amplified using a KAPA HiFi Hotstart PCR kit (Kapa Biosystems, Wilmington, NC, USA) with 5 uM primers. PCR products were subjected to 2% agarose gel electrophoresis and purified using AMPure XP magnetic beads (Beckman Coulter, Wycombe, UK). The qualities of purified amplicons were measured using a Bioanalyzer 2100 (Agilent, Santa Clara, CA, USA). Secondary amplification was then performed over 8 cycles to attach Illumina Nextera barcodes (Illumina, Inc., San Diego, CA, USA) using i5 forward and i7 reverse primer. Amplified products were purified using AMpure XP magnetic beads (Beckman Coulter, Brea, CA, USA) according to the manufacturer’s protocol. Purified amplicons were quantified using a QuantiFluor ONE dsDNA System (Promega, Madison, MI, USA). Amplicon sizes and qualities were evaluated using a Bioanalyzer 2100 (Agilent, Santa Clara, CA, USA). Pooled libraries were sequenced using an illumina MiSeq instrument and the MiSeq v3 Reagent Kit (Illumina, Inc., San Diego, CA, USA).

### 2.5. Data Analysis

An analysis of the raw data of the 16S rRNA gene sequences was processed using the QIIME (v1.9.1) bioinformatics pipeline. Using qualified sequences (paired-end, Phred ≥ Q20), the operational taxonomic units (OTUs) were assigned based on an open-reference picking method using 97% identity to entries in the Greengenes database (V13_8) using UCLUST. Alpha diversity was calculated using phylogenetic distances and numbers of observed OTUs. For beta diversity comparison between groups, UniFrac distances were evaluated. The comparisons between groups were analyzed using Welch’s *t*-test; a *p* value was assessed as significant when <0.05.

## 3. Results

### 3.1. Baseline Clinical Characteristics

Twelve patients were enrolled in the study group, and a follow-up biopsy was performed on five of them. The age range of patients was 43–85 years (median, 67.5 years), and six were men. Body mass indices ranged from 17.4–27.6 (median, 21 kg/m²). Ulcer locations and stages are listed in Table 1. Ulcers were located mainly in the antrum and lower stomach body. Ulcer stages were evaluated using the Sakita-Miwa classification [29]. All gastric ulcers were confirmed to be in the healing process during follow-up endoscopy.

### 3.2. Bacterial Composition in Duodenum

**Figure 2:**
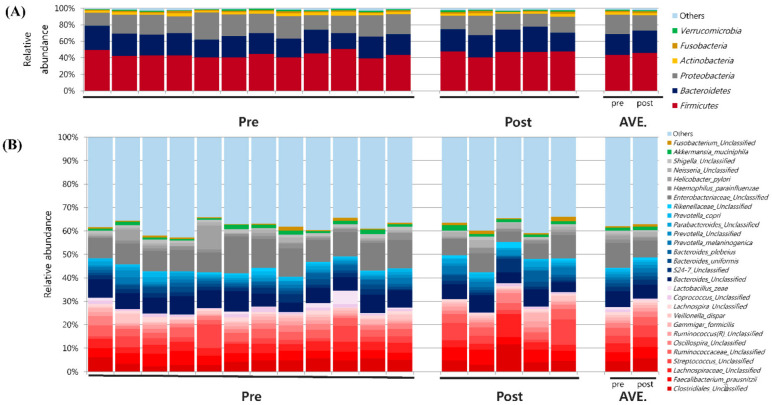
Microbial analysis of second duodenal portion mucosal samples obtained from gastric ulcer patients. (**A**) Microbiome composition at phylum level, and (**B**) at genus level.

At the phylum level, the predominant bacteria groups were five in the pre- and post-PPI group at an average percentage composition of ≥1% of total microbiota. *Firmicutes* predominated in pre- and post-treatment samples (average percentage composition, pre-PPI: 43.5% vs. post PPI: 45.9%). Percentages of *Bacteroidetes* (25.4% vs. 26.9%) and *Proteobacteria* (23.4% vs. 18.8 %) were also high, followed by *Actinobacteria* (3.0% vs. 2.5%) and *Fusobacteria* (1.8% vs. 2.1%) (Figure 2A). At the genus level, the family *Clostridiaceae* (phylum *Firmicutes*) was present at the highest percentage (13.4% vs. 16.2%), followed by *Faecalibacterium* (11.9% vs. 13.7%) and *Lachnospiraceae* (11.3% vs. 11.8%). In the phylum *Bacteroidetes*, the percentage of *Bacteroides* was highest (55.4% vs. 51.3%), followed by *Prevotella* (17.9% vs. 21.3%) (Figure 2B).

### 3.3. Bacterial Community Diversity Pre- vs. Post-PPI Treatment

**Figure 3:**
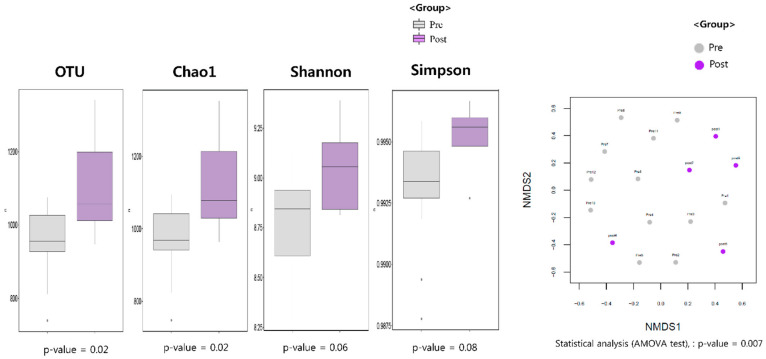
(**A**) Microbial richness assessed by number of OTUs, Chao1, and biodiversity index, including Shannon, Simpson indices (**B**) Beta diversity visualized using non-metric multidimensional scaling (NMDS) plot with Bray–Curtis dissimilarity distances.

The number of OTUs observed in the samples was significantly higher after PPI treatment (*p* = 0.02). Alpha diversity metrics, the Chao1 estimator, and Shannon diversity indices were used to evaluate species abundances, and the Simpson index was used to evaluate species evenness. Obviously, there were significant differences in the Chao1 index pre- and post-PPI treatment (*p* = 0.02, Figure 3A). This shows that bacterial community diversity increased after PPI treatment with statistical significance. Significance was determined by analysis-of-variance (ANOVA). The Shannon and Simpson indices for the pre-PPI group were lower than for the post-PPI group, indicating lower bacterial community diversity. However, there was no statistically significant value. (Figure 3A, *p* = 0.06 and 0.08, respectively). We also assessed the significance of bacterial community composition (i.e., beta diversity) between individuals using NMDS plots on rank-order Bray–Curtis distances. As shown in Figure 3B, clustering by sample showed significant differences in beta diversity between the pre- and post-treatment group (*p* = 0.007).

**Figure 4:**
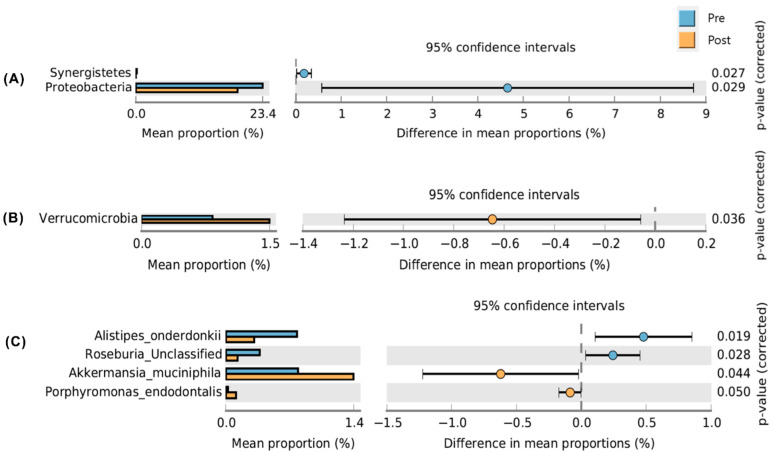
Welch’s *t*-test to compare the profiles at the phylum level (**A**) genus level (**B**) and species level (**C**).

Welch’s *t*-test was used to compare profiles at the phylum level and showed that the abundance of *Proteobacteria* was significantly decreased (*p* = 0.029), and that of *Verrucomicrobia* was increased in the post-PPI group (*p* = 0.036) (Figure 4A,B). At the genus level (Figure 4B), Welch’s *t*-test showed that the abundances of *Enterococcaceae*, *Coprococcus*, *Enterobacteriaceae*, and *Synergistes* were significantly decreased, and those of *Porphyromonas* and *Akkermansia* were significantly increased after treatment (Figure 4C).

## 4. Discussion

In this prospective study, we compared and analyzed the duodenal microbiomes of gastric ulcer patients treated with a PPI. The study provides initial data on how the diversity of the duodenal microbiome in gastric ulcer patients is altered by PPI treatment. A duodenal biopsy was performed during gastroduodenoscopy in the second portion of the duodenum before PPI treatment and 4 weeks after treatment. Significant differences in duodenal microbiomes were observed after treatment. In particular, Chao1 index values were significantly increased by treatment, which means that duodenal microbiome richness was significantly increased by PPI treatment. Beta diversity was also significantly altered by PPI treatment.

The small intestine, including the duodenum, unlike the stomach and colon, has only one type of surface mucus, and this mucus is unattached and easily removed [30]. In addition, the duodenum contains a high concentration of bile acid, which has antimicrobial effects, so it is not a good environment for bacteria. Accordingly, the upper two-thirds of the small intestine have a much lower bacterial concentration than the colon (10^3–4^ vs. 10^11–12^ microorganisms per mL) [31], which in part explains the lack of study of the duodenal microbiome. In previous studies, *Lactobacillus* sp., *Escherichia coli*, and *Enterococci* were found to be the predominant species in the duodenum and jejunum [31,32]. However, because these analyses were performed on the microbiome of the small intestine, including the jejunum, the results obtained cannot explain the characteristics of the duodenum microbiome. Therefore, the present analysis of the characteristics of the microbiome of duodenal mucosa contributes to current knowledge. In this study, *Firmicutes* was most abundant (43.5 %), followed by *Bacteroidetes* (25.4%), *Proteobacteria* (23.4%), *Acinetobacteria* (3.0%), and *Fusobacteria* (1.8%), which concurs with the results of recent studies on duodenal fluid [28,33,34].

We identified significant differences after PPI treatment at the genus and species levels. The diversity and richness of the mucosa-associated microbiome were greater after treatment. The dominant difference in the bacterial composition in the pre-PPI treatment group was observed in *Alistipes_onderdonkii* and *Roseburia_unclassified*. *Alistipes_onderdonkii* has been suggested to have protective effects against some diseases, including liver fibrosis, colitis, and cardiovascular disease, and against cancer immunotherapy [35]. *Roseburia spp*. are known to produce short-chain fatty acids, specifically butyrate, which affects colon motility, the maintenance of immunity, and anti-inflammatory properties [36]. In contrast, the dominant difference in bacterial composition in the PPI-treated group was the presence of *Akkermansia mucinophila* and *Porphyromonas endodontalis*. Previous studies have shown that *Porphyromonas endodontalis* is associated with periodontitis [37,38]. *A. mucinophilia* is considered a gut symbiont associated with numerous health-enhancing effects [39]. These observed changes indicate that PPI treatment induces duodenal dysbiosis.

In this study, the average percentage and proportion of *Akkermansia* at the genus level increased significantly after PPI treatment, and the average proportion of *Akkermansia muciniphila* also increased significantly. *A. muciniphila* has been reported to have the ability to obtain nutrients by breaking down mucin and modulating the inflammatory response in a mouse model [40,41]. Also, the abundance of *A. muciniphila* has been found to be inversely proportional to the severity of appendicitis, as well as being present in lower amounts in patients with inflammatory bowel disease, suggesting an association with human health problems [42,43]. *A. muciniphila* is a viable strain due to its decomposing mucin in the outer layer of mucus secreted by goblet cells in gastric mucosa [44]. In patients with gastric ulcer, the healing process begins within 2–3 days after ulceration. After re-epithelialization begins at the ulcer margin, granulated tissue of the ulcer base restores epithelial continuity [45]. Furthermore, the mucus produced during re-epithelialization can become the medium for *A. muciniphila* growth, and the host may benefit due to the anti-inflammatory effects of the metabolites produced. This was interpreted as a result opposite to the detrimental effect of PPI-induced bacterial overgrowth in gastric ulcer patients. Conversely, the increase in *A. muciniphila* may have effects such as increased permeability and duodenal dysbiosis, as in small intestinal bacterial overgrowth, so further, subsequent studies are needed [46,47].

In general, dysbiosis means that the richness and abundance of the gut microbiome are decreased, and the diversity also tends to decrease. However, all three increased after PPI treatment. The colon contains more than 10⁷ times the number of microorganisms found in the duodenum, and the bacteria present produce metabolites and interact with host immune cells. Thus, dysbiosis in the colon is defined as a decrease in bacterial diversity. On the other hand, it is difficult to apply the same definition to the duodenum because the number of microorganisms is relatively small, as food materials pass through the duodenum quickly, which means the duodenal environment militates against bacterial survival. Further studies are needed to define duodenal dysbiosis accurately.

The peculiarity of our study is that it is the result of analyzing a biopsy specimen and considering the characteristics of the duodenal mucosa. In addition, the results of this study are consistent with the results of recent studies. PPIs induce duodenal bacterial overgrowth and have been shown to be associated with the development of severe acute pancreatitis and recurrent cholelithiasis, and our study suggests important microbiome changes in these developments after PPI use [48,49].

Despite its prospective design, this study has some limitations. First, the number of patients included in the evaluation was small. Second, healthy controls with no upper gastrointestinal abnormalities such as gastric ulcer were not included in this study. Despite the above two limitations, we consider the study meaningful, as it provides statistically derived information on the effects of a PPI on the duodenal microbiome in a background of gastric ulcer. Third, there was no analysis of *H. pylori* infection and eradication, which can change the microbiome of the stomach and duodenum. Because this study was designed to analyze changes in the microbiome of the duodenum according to the use of PPIs, it was not handled. Nevertheless, it is necessary to evaluate the effect of *H. pylori* infection and eradication, so a large-scale subsequent study should be conducted in the future. Fourth, this study showed changes in duodenal bacterial compositions but did not explain how the composition of the bacteria affects host health, including the gut–brain axis. A well-designed large-scale study is needed to explore this issue.

In conclusion, this study showed that species abundance increased, and microbiome compositions changed significantly after PPI treatment in the duodenum of gastric ulcer patients. PPI treatment decreased the abundances of the beneficial bacteria *Alisipes onderodonkii* and *Roseburia*, and it increased the abundance of the known pathogen *Porphyromonas endodontalis*. Furthermore, the abundance of *Akkermansia mucinophilia* increased after PPI treatment, which we suggest was related to increased duodenal mucus and gastric ulcer healing with an increase in gastric pH. These findings lead us to speculate that PPI use is associated with the development of dysbiosis in the duodenal microbiome.

## Figures and Tables

**Table 1:** The patients’ information.

Groups	Sex	Age	BMI (kg/m^2^)	Ulcer Location	Ulcer Stage	Follow-Up Biopsy
A1	M	68	27.6	Antrum	A2	YES
A2	M	70	19.7	Low body	A2	NO
A3	F	68	19.7	Mid body	A2	NO
A4	F	72	24.4	Antrum	H1	YES
A5	M	63	27.3	Antrum	A2	YES
A6	M	43	17.4	Antrum	H1	NO
A7	F	66	25.8	Antrum and Body	A2	YES
A8	M	61	21.5	Antrum	H1	NO
A9	F	67	20.5	Antrum	A2	YES
A10	F	77	18.3	Antrum	H1	NO
A11	M	67	19.3	Antrum	A1	NO
A12	M	85	27.6	Low body	H1	NO

## Data Availability

Not applicable.
